# A new technique for minimally invasive irreversible electroporation of tumors in the head and body of the pancreas

**DOI:** 10.1007/s00464-016-5173-6

**Published:** 2016-08-29

**Authors:** David Stillström, Henrik Nilsson, Michaela Jesse, Matthias Peterhans, Eduard Jonas, Jacob Freedman

**Affiliations:** 1 Department of Clinical Sciences Danderyd Hospital, Karolinska Institutet, Stockholm, Sweden; 2 Department of Surgery, Clintec, Karolinska Institute, Karolinska University Hospital, Stockholm, Sweden; 3 MeVis Medical Solutions AG, Bremen, Germany; 4 CAScination AG, Bern, Switzerland; 5 Surgical Gastroenterology Unit, Department of Surgery, Groote Schuur Hospital, University of Cape Town Health Sciences Faculty, Cape Town, South Africa

**Keywords:** Computer-assisted navigation, Pancreas, Adenocarcinoma, Irreversible electroporation, IRE, Laparoscopy

## Abstract

**Background:**

Palliative irreversible electroporation of pancreatic adenocarcinomas is rapidly gaining in interest since a large proportion of these patients cannot be radically resected.

**Methods:**

This is a description of a minimally invasive approach to irreversible electroporation of pancreatic tumors using computer-assisted navigation, laparoscopy and laparoscopic ultrasound to correctly guide electrodes into the tissue.

**Results:**

The procedure is presented.

**Conclusion:**

Minimally invasive irreversible electroporation of pancreatic tumors through computer-assisted navigation of needles during laparoscopy is a feasible and accurate approach.

**Electronic supplementary material:**

The online version of this article (doi:10.1007/s00464-016-5173-6) contains supplementary material, which is available to authorized users.

## Background

Pancreatic cancer accounts for 4 % of cancer deaths worldwide and is on the trajectory of becoming the second leading cause of cancer-related deaths in the USA by 2030. Fifty percent of patients will have a disseminated disease at the time of diagnosis [1]. In patients with localized tumors, approximately 20 % are resectable with a disappointing 5-year survival of 20–25 % being reported [2, 3]. The remaining patients receive oncologic treatment (downstaging or palliative) or best supportive care, with a dismal 5-year survival of around 2 % [4].

Resection of tumors in the head of the pancreas involves major surgery with resection of the pancreas, duodenum, distal bile duct and often part of the stomach, and in selected cases, resection of vascular structures involved [5]. Postoperative morbidity is high, and problems such as delayed gastric emptying are common [6].

Alternative means for local treatment has been investigated, including radiation therapy and a variety of thermal and non-thermal local ablation techniques generally with discouraging results [7–9]. Thermal options are problematic because of the proximity of vital vasculature structures and pancreatic and bile ducts, all being sensitive to thermal ablation. Irreversible electroporation (IRE) is a relatively new method for local treatment, achieving cell death by the application of short electrical pulses (millisecond pulses with field strengths of up to 1500 V/cm) while causing minimal thermal effects on connective tissue. Vascular and ductal structures are there for generally spared. Promising results have been reported for IRE as an adjuvant to chemotherapy, almost doubling median survival in palliative patients and increasing resection margins in resections with curative intent [10]. The IRE electrodes are applied during open surgery or percutaneously using ultrasound guidance. Problems with these approaches include trauma caused by the laparotomy and avoiding damage to surrounding organs, especially with the percutaneous approach.

In an effort to address these issues, a laparoscopic approach has been developed using 3D mapping of relevant structures, computer-assisted navigation and laparoscopic ultrasound.

## Methods

Patients were all presented at a pancreas multidisciplinary team (MDT) conference, and the possibility of curative surgery was ruled out. They were referred for palliative chemotherapy and on patient demand assessed for IRE ablation. In patients, deemed suitable for treatment, the experimental nature of the treatment was explained and written consent obtained.

Only patients with localized disease without any signs of metastases were included. Procedures were performed under general anesthesia with a complete neuromuscular block to reduce muscle contractions caused by the strong electrical pulses administered. A 3D anatomical map, constructed from a 3-phase preoperative CT scan using dedicated software (MeVis AG, Bremen, Germany), was loaded into the CAS-One navigation system (Cascination AG, Bern, Switzerland), consisting of a computer with a stereotactic infrared camera and dedicated navigational software developed for open liver surgery [11]. Using ultrasonic energy (Harmonic ACE, Ethicon Endo-surgery, USA), the gastrocolic ligament was opened along the greater curvature of the stomach right up to the pylorus. The stomach was displaced cephalad, and the ventral aspect of the pancreas visualized. Four-point matching with the 3D model was performed using a rigid pointing instrument for acquiring anatomical structures around the tumor which were directly visible or identifiable on ultrasound. The formation pattern for the IRE needles was designed based on tumor-specific properties with consideration of surrounding structures. Needles were inserted into the abdominal cavity through coaxial stabilizing needles and advanced into the pancreatic tissue at a point defined using the rigid pointer and to a depth indicated by the navigational software that recognizes reflective spheres on a marker shield attached to the handle of the IRE needle. Laparoscopic ultrasound was used to ascertain optimal needle positioning, for example, by visualizing the superior mesenteric artery and placing needles on each side to ablate tumorous tissue around the artery.

**Fig. 1:**
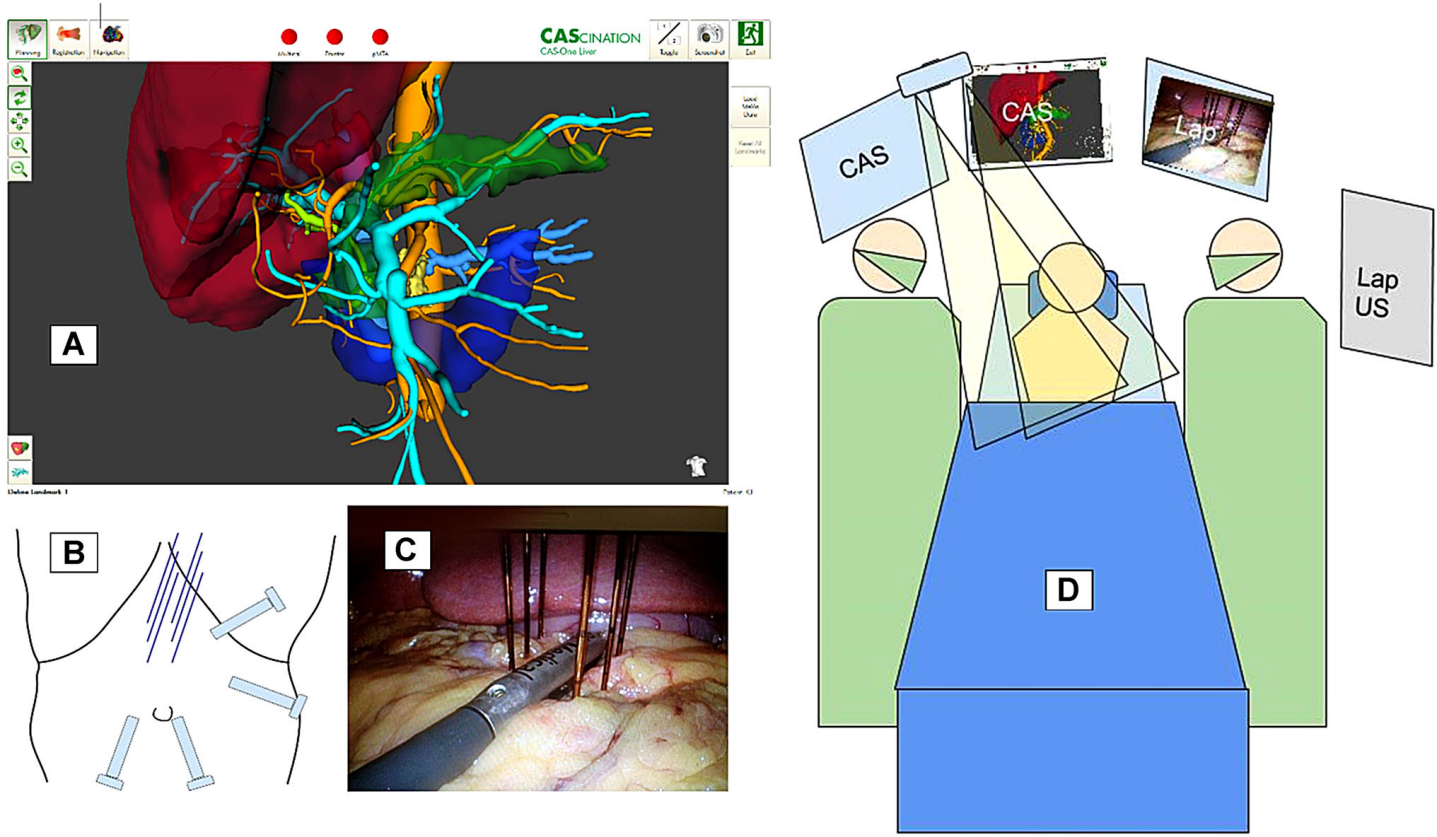
Patient setup with CAS-One on the patients right with twin infrared cameras and laparoscopic screen and ultrasound screen on the left. **A** On-screen image of rendered abdominal structures in the MeVis model. **B** Laparoscopic port placements and site of IRE needle entries. **C** Laparoscopic view of laparoscopic ultrasound device aligned over the superior mesenteric artery and IRE needles inserted in a 2 × 3 fashion to surround the tumor. **D** Setup of surgeons and monitors

After application of the calculated ablation energy, needles were removed under direct visual control ensuring hemostasis (Fig. 1).

## Results

### Patient 1

**Fig. 2:**
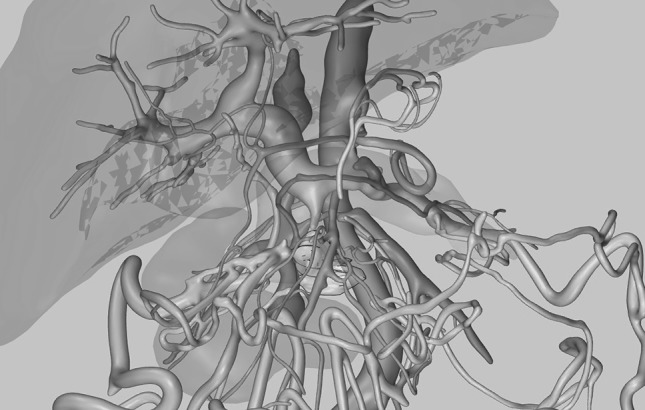
3D reconstruction of preoperative CT-scan for patient 1

A 75-year-old woman with an initially 30-mm pancreatic adenocarcinoma surrounding the superior mesenteric artery was treated after a response on chemotherapy (10-mm shrinkage) was achieved. Surgery took 190 min, and the patient was discharged after 12 days. The postoperative period was complicated by an acute fluid collection which was drained percutaneously and vanished. Postoperative imaging after 8 months showed a stable 25-mm lesion and up to 8-mm-large lymph nodes in the vicinity, a situation that has remained unchanged for the subsequent 6 months (Fig. 2).

### Patient 2

**Fig. 3:**
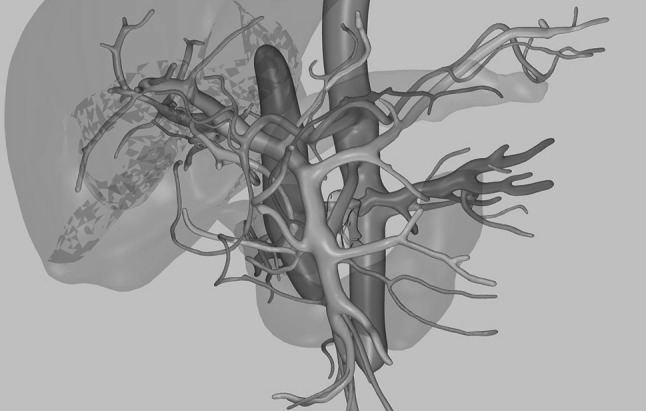
3D reconstruction of preoperative CT-scan for patient 2

A 64-year-old man with a 35-mm pancreatic adenocarcinoma surrounding the superior mesenteric artery was treated after 1-year chemotherapy treatment resulted in stable disease. Surgery took 156 min, and the patient was discharged after 5 days. The postoperative course was uneventful. Postoperative imaging at 6 months showed a diffused perivascular infiltration with subsequent growth observed at the tumor site, measuring 30 × 80 mm at one-year follow-up. After 14 months, the patient is still alive and under palliative care (Fig. 3).

### Patient 3

**Fig. 4:**
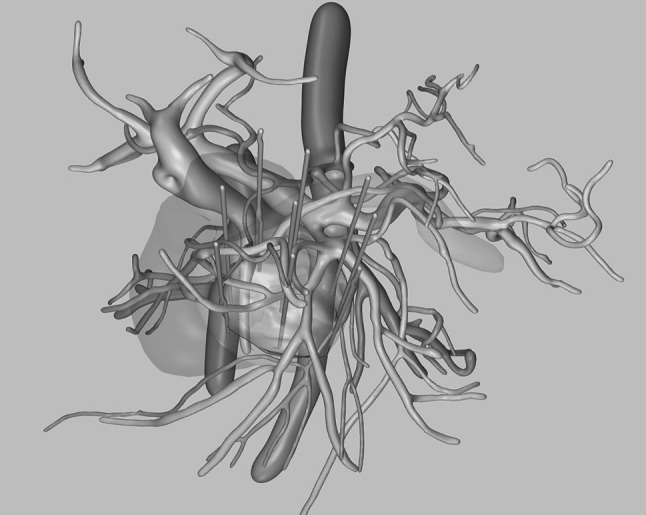
3D reconstruction of preoperative CT-scan for patient 3

A 78-year-old woman with a 50 × 45 mm pancreatic adenocarcinoma surrounding the superior mesenteric artery, engaging most of the uncinate process, was treated. The duration of surgery was 282 min of which the treatment took 160 min. The postoperative period was complicated by pancreatitis with acute fluid collections and spondylodiscitis affecting the epidural space. On day 6, there was a small duodenal perforation that was successfully covered by a covered stent. Postoperative imaging has shown complete ablation of the tumor, and the patient could after 100 days of hospice care return home. After 146 days, the patient is cared for in her home with minimal discomfort, but a new CT scan has showed recurrent tumor, and palliative chemotherapy is planned (Fig. 4).

## Discussion

We present a novel method for minimally invasive irreversible electroporation in the palliative treatment of pancreatic tumors. The present work describes a workflow for virtual 3D treatment planning and computer-assisted navigation for guidance of electrode placement, which was successfully implemented on three patients. We believe that computer-assisted navigation improves orientation in the complex vascular settings, enabling IRE treatment with a laparoscopic approach.

Although this technique offers minimal trauma for the access, the ablative treatment in itself can cause severe local trauma to the tumor and surrounding pancreatic tissue with resulting secondary pancreatitis associated with postoperative pain, fluid collections and pseudocyst formation that need to be managed as in any pancreatitis.

A randomized controlled study examining the efficacy of the addition of minimally invasive IRE to standard chemotherapy of primarily inoperable localized pancreatic tumors is planned.

A 3D-PDF file is included and is the 3D reconstruction for the third patient with the resulting ablated volume overlaid and an approximation of the needle configuration used. The file must be opened in Adobe Acrobat Reader to work properly.

## Electronic supplementary material

Below is the link to the electronic supplementary material.

Supplementary material 1 (PDF 4420 kb)
