# Supplementary material for: A new technique for minimally invasive irreversible electroporation of tumors in the head and body of the pancreas
Source: Surg Endosc. 2016 Aug 29;31(4):1982–5. doi: 10.1007/s00464-016-5173-6 (PMC5346119; doi:10.1007/s00464-016-5173-6)
Supplement: Supplementary file 1 — Supplementary material 1 (PDF 4420 kb) [file 464_2016_5173_MOESM1_ESM.pdf]

MeVis Distant Services - Liver Report

Patient Data: anonymizedID: anonymized

Patient Data: anonymized
ID: anonymized

Warning:

MDS is a purely technical service for education, publication and presentation purposes only; it is not a medical product and should not be used for clinical decisions. MMS does not provide medical reports, medical diagnoses or recommendations nor does MMS review or evaluate reports in medical respect.

Accept

Decline

Report creation tool: MeVis ReGen (GUI Version), Version 2.1.4.0
Report data collected by: MeVis ReGen (MDS Liver Data Input Driver), Version 2.0.7.1
Report document created by: MeVis ReGen (MDS 3D-PDF Output Driver), Version 2.0.4.3
